# Retraction: Over-Expression of Superoxide Dismutase Ameliorates Cr(VI) Induced Adverse Effects via Modulating Cellular Immune System of *Drosophila melanogaster*

**DOI:** 10.1371/journal.pone.0225470

**Published:** 2019-11-13

**Authors:** The PLOS ONE Editors

After publication of this article [1], concerns were raised about similarities between the following figure panels:

Confocal microscopy images:

- Fig 1D Control and 20.0 μg/ml Mo(VI) panels appear to contain a region of overlap
- Part of Fig 1D, 20.0 μg/ml Cr(VI) appears similar to Fig 9D2, Control panel
- Part of Fig 1D, 20.0 μg/ml Mo(VI) appears similar to Fig 9A2, Control panel
- Part of Fig 6A2 (20.0 μg/ml Cr(VI) + L-NAME, *Oregon R*^+^) appears to overlap with an area in Fig 9A2 (*HUAS-SodRNAi*)

Flow cytometry dot plot images:

- Fig 3B(a), 6B2(a), and 9C2(a) appear similar
- Fig 6B2(b) and 9C2(b) appear similar
- Fig 3B(b) is similar to Fig 9C2(c)

In regard to the confocal microscopy concerns, the authors noted that incorrect representative images were used in the figures and that the quantification data reported in the article were not obtained from those images. They provided updated figure panels for Figs 1D, 6A2, 9A2, and 9D2 in which data of concern were replaced with images that were reportedly obtained during the original experiment. A *PLOS ONE* Academic Editor reviewed these updated figures and confirmed that they support the results reported in the original publication.

In response to the flow cytometry issues, the authors explained that the flow cytometry experiments were outsourced to another institution, as noted within the Acknowledgements of the published article. The authors provided some raw flow cytometry data as well as dot plots to support the figures of concern and claimed that the dot plots reported results for independent replicates of each experiment. In our editorial assessment of the files, we identified instances in which there appeared to be overlap between dot plots provided for different experiments. Academic Editors with expertise in flow cytometry reviewed the data files, confirmed that in several instances the same data were used to represent different experimental results, and advised that data reported in quadrant 2 (Q2) of most dot plots are indicative of autofluorescence. The Academic Editors further advised that the gating strategy was not adequately reported.

During follow-up discussions the authors provided a second set of flow cytometry data files but they did not clarify the concerns with the original dataset.

The *PLOS ONE* Editors retract this article in light of the above issues, which call into question the validity and reliability of the results.

RCM and DKC disagree with retraction. PP, AKS, and MZA did not respond or could not be reached.
